# Cemented hemiarthroplasty versus proximal femoral nail antirotation in the management of intertrochanteric femoral fractures in the elderly: a case control study

**DOI:** 10.1186/s12891-021-04586-x

**Published:** 2021-10-05

**Authors:** Zhuangzhuang Jin, Shuoyan Xu, Yue Yang, Yingliang Wei, Yicheng Tian, Ziyuan Wang, Lunhao Bai

**Affiliations:** 1 China Medical University, Shenyang, Liaoning China; 2 Department of Emergence Medicine, Shengjing Hospital Affiliated China Medical University, Shenyang, Liaoning China; 3 Department of Nuclear medicine, The First Hospital Affiliated China Medical University, Shenyang, Liaoning China; 4 Department of Orthopedics, Shengjing Hospital Affiliated China Medical University, Heping District, 110004 Liaoning China

**Keywords:** Clinical outcome, Hemiarthroplasty, Hip fracture, Intertrochanteric fracture, PFNA, Prognosis

## Abstract

**Background:**

The treatment for intertrochanteric femoral fractures (IFF) among the elderly has been a controversial topic. Hemiarthroplasty (HA) and proximal femoral nail antirotation (PFNA) have their own advantages in the management of IFF. Hence, this study aims to compare and analyze differences in the effectiveness of both procedures on IFF among the elderly.

**Methods:**

Overall, 99 patients (81.09 ± 8.29 years; 68 women) underwent HA or PFNA from January 2016 to May 2020. IFF were classified according to the Arbeitsgemeins für Osteosynthesefragen (AO) classification. The difference in underlying diseases, the American Society of Anesthesiologists (ASA) grade, Singh index, Harris scores, surgical time, intraoperative bleeding, postoperative blood test results, postoperative number of days to partially bearing weight, and survival outcomes were analyzed. Postoperative follow-ups were performed every 3 months.

**Results:**

There was no significant difference in the AO classification, underlying diseases, ASA grade, Singh index, surgical time, and survival outcomes of the HA (45 patients) group and PFNA group (54 patients). The HA group was associated with earlier partial weight-bearing (HA: 4 [2 ~ 4.5] days, PFNA: 10 [8~14] days). It also had a higher total Harris score than the PFNA group at the 6-month follow-up visit (HA: 86.8 [81.90 ~ 90.23], PFNA: 83.48 [75.13 ~ 88.23]). Harris scores decreased more in patients aged ≥90 years in the PFNA group than in the HA group. The postoperative stress recovery rate in the HA group was faster based on postoperative blood test results.

**Conclusions:**

PFNA and HA have good therapeutic effects in the treatment of IFF. The advantages of HA were reflected in short-term weight bearing, faster recovery from stress, and better joint function in the long term. This advantage is more obvious in the patient population aged over 90 years. Therefore, we suggest that surgeons should consider the benefit of HA in the treatment of IFF among the elderly.

**Trial registration:**

Chinese Clinical Trial Registry, ChiCTR2000035814. Registered 17 August 2020, https://www.chictr.org.cn/showproj.aspx?proj=57083

## Introduction

The incidence of intertrochanteric femoral fractures (IFF) among the elderly is increasing [1]. IFF are the main type of proximal femur fractures, which account for the occurrence of bedrest-related complications, such as deep vein thrombosis, urinary tract infection, pulmonary infection, gastrointestinal disorders, and decubitus ulcers. The mortality rate within 1 year of the incidence of IFF is reportedly as high as 20% [2]. Most elderly patients have various underlying diseases, including osteoporosis, diabetes, cardiovascular or chronic respiratory diseases, which increase the risk of surgery and the difficulty of rehabilitation.

There are two main approaches to surgical repair of IFF: proximal femoral nail antirotation (PFNA) and hemiarthroplasty (HA) [2, 3]. PFNA was developed by the Arbeitsgemeins für Osteosynthesefragen (AO) Foundation, which is a nonprofit global network of surgeons for the surgical management of musculoskeletal disorders. Most researchers advocate the use of PFNA in the treatment of IFF and in senile Chinese patients [4, 5]. Biomechanical tests have shown that PFNA has a better effect on anti-rotation and anti-inversion than traditional internal fixation [6]. The distal-locking screws of PFNA can maintain fracture length, prevent limb shortening, and increase fracture stability [7]. However, some elderly patients experienced a longer period of bed rest after undergoing PFNA. Because of the poor bone condition of the elderly, screw cutting may occur after surgery. The revision surgery after failed PFNA procedures may be unbearable for some elderly patients. The distal-locking screw of PFNA can cause pain, femoral cortical erosion, or fracture around the screw [8]. HA has gained acceptance for the treatment of IFF, and its efficacy has been confirmed [9–11]. HA has shown a marked advantage for early postoperative weight-bearing and reduction of immobility-related complications. It also has advantages in patient stress rehabilitation and joint mobility. However, potential problems, such as excessive blood loss, partial bone defects, and joint capsule scar adhesion, still exist [12].

Both PFNA and HA have advantages and disadvantages; however, there has been little consensus on the best choice for the surgical management of IFF. Therefore, to compare their curative effect, especially the basic preoperative conditions, surgical data, stress recovery speed during postoperative hospitalization, functional outcome, and long-term mortality, a case-control study was conducted at the Shengjing Hospital Affiliated China Medical University.

## Patients and methods

### Inclusion and exclusion criteria

This trial was registered in the Chinese Clinical Trial Registry (identifier ChiCTR2000035814; registration date August 17, 2020, https://www.chictr.org.cn/showproj.aspx?proj=57083). Overall, 162 patients diagnosed with IFF underwent surgical procedures from January 1, 2016 to May 31, 2020. This study included 99 elderly (aged ≥60 years) patients with IFF. All patients underwent HA or PFNA for the first time. All surgeries were performed by the corresponding author of this study. The exclusion criteria were as follows: (1) age < 60 years; (2) open fracture, pathological fracture, or previous IFF; (3) IFF with multiple injuries (polytrauma); (4) local or systemic infection; (5) lack of informed consent; (6) bone metastases; (7) loss to follow-up. This study was approved by the ethics committee of the Shenjing Hospital Affiliated China Medical University. Before the procedure, we fully explained the advantages and disadvantages of the two procedures (HA and PFNA) including the difference in surgical cost and recovery. The patient or authorized person had the right to decide on the surgical plan. Written informed consent was obtained from all patients before surgery. This study followed the newest Strengthening the Reporting of Cohort Studies in Surgery guidelines [13].

### Patient information

General information, including clinical history, surgical record, nursing record, and imaging data, were collected from the hospital information system. Anesthesia and surgical risk was determined preoperatively by the American Society of Anesthesiologists (ASA) score. Assessment of the bone quality was based on the Singh index. The fracture type was defined according to the AO classification.

### PFNA technique

After successful administration of the anesthesia in PFNA, the affected limb was internally rotated, adducted, and fixed by traction. Closed reduction of the fracture was performed under C-arm fluoroscopy. The surgery began after routine disinfection and placement of a sterile sheet and protective film for the incision. A 5-cm longitudinal incision was made 2 cm from the apex of the femoral trochanter. The apex of the femoral trochanter was exposed following the separation of subcutaneous tissues, fascia, and muscle. Using C-arm fluoroscopy, a guidewire was inserted through the tip of the greater trochanter into the medullary cavity. The medullary cavity was expanded using an electric drill along the direction of the needle. Subsequently, the main screw of PFNA was carefully inserted. The anti-rotation screw was inserted below the centerline of the femoral neck and measured satisfactorily under fluoroscopy. A hip screw of appropriate length was then inserted and screwed.

### Cemented HA technique

The cemented HA technique was performed in the HA group. A posterolateral approach to the hip joint was performed after establishing a sterile environment. The subcutaneous tissues, fascia, and muscle were separated layer-by-layer. The fascia was incised on the posterior edge of the greater trochanter after blunt dissection of the gluteus maximus along the direction of the muscle fibers. The lower limb was internally rotated, and a series of muscles, including the piriformis and internal obturator, were cut to expose the posterior aspect of the hip joint. The articular capsule was split into a tongue shape, and the hip was intentionally dislocated. Osteotomy was performed at the femoral neck to completely remove the femoral head. The medullary cavity was opened and expanded by an electric drill at the proximal femur. The prosthesis was used to check the matching degree with the medullary cavity, which was lavaged with normal saline, and the cemented femoral stem was installed quickly. The femoral head prosthesis was installed, and the hip joint was relocated to test the matching degree, after which the bipolar cup was fixed and the degree of motion and stability of the hip joint were affirmed. The surgical window was flushed with normal saline, and hemostasis was completed by suture ligation or electrocautery. Part of the joint capsule and severed muscle, including the piriformis and obturator internal, were sutured with nonabsorbable sutures, and the incision was closed layer-by-layer.

### Postoperative rehabilitation and follow-up

The vital signs, including blood pressure, pulse, respiration, heart rhythm, and blood oxygen saturation, were closely monitored during the entire surgery and 24 h after surgery. All patients received comprehensive postoperative care according to the basic protocol, including antibiotics, anti-coagulants, analgesic agents, and incision care. The patients in both groups were asked to perform quadriceps muscle contraction and relaxation to avoid muscle atrophy from postoperative day 2. Patients were also encouraged to walk without weight bearing. Subsequently, we recorded the time when they begin to bear partial weight. Weight-bearing gradually increased until patients could completely support their entire body weight.

The first follow-up visit was scheduled for 6 weeks after the surgery, and patients were referred for imaging at our hospital or at their local hospital. Three months after the surgery, the extent of healing of the fracture or the condition of the prosthesis was evaluated based on clinical manifestations, physical examination, and imaging data. Recovery of hip joint function was evaluated by the postoperative Harris hip score with a standard questionnaire at the 6-month follow-up [14]. After the fracture healed, follow-ups were scheduled every 3 months to record survival information.

### Statistical analyses

SPSS 21.0 software (IBM Corp., Armonk, NY, USA) was used for statistical analyses. All data were evaluated for homogeneity of variance and normality using Shapiro-Wilk test and Levene’s test, respectively. T-tests were used for continuous variables with homogeneity of variance and normality, which was expressed as mean ± SD. Otherwise, Wilcoxon rank-sum tests were used, and data were expressed as medians with interquartile range (IQR [25% quartile to 75% quartile]). Counting data were analyzed using the Wilcoxon rank-sum test or Chi-square test, which were expressed as median (IQR [25% quartile to 75% quartile]). Probability (*P*) value < 0.05 was considered statistically significant.

## Results

### No difference in baseline characteristics

**Fig. 1:**
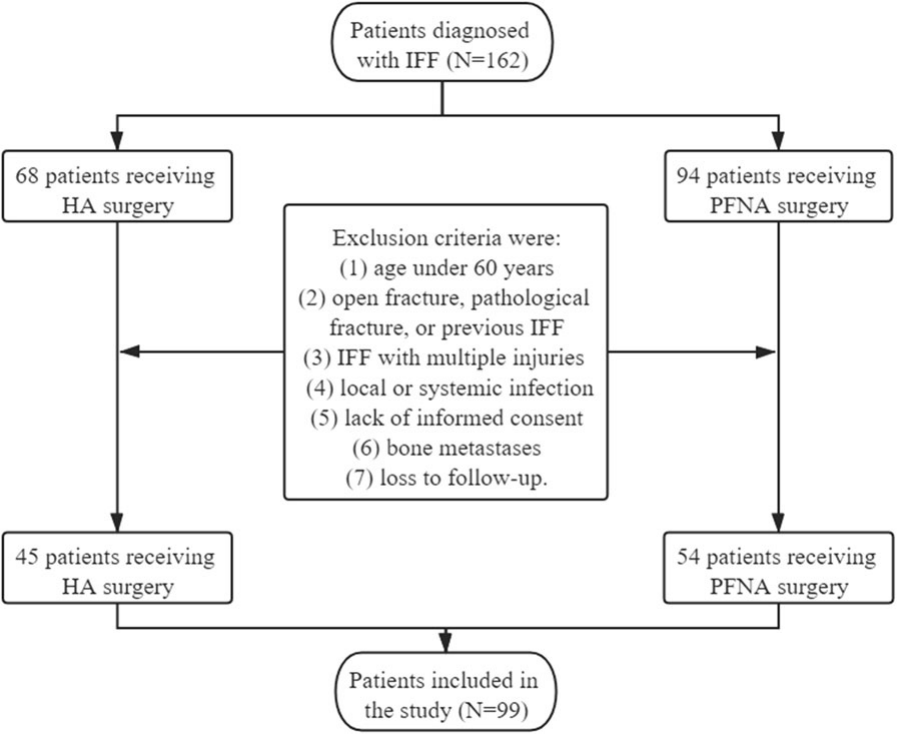
Flow chart for the inclusion of subjects. Abbreviations: HA, hemiarthroplasty; IFF, intertrochanteric femoral fracture; PFNA, proximal femoral nail antirotation

Figure 1 shows the number of patients and details of their enrollment. During the selected period, 162 patients with IFF underwent surgery, of which 68 underwent HA and 94 underwent PFNA. After adhering to the exclusion criteria, 45 patients were included in the HA group and 54 patients were included in the PFNA group. Overall, 99 patients (81.09 ± 8.29 years; 68 women [68.7%]) were evaluated. Table 1 provides an overview of the baseline characteristics of both groups. There was no difference in patient baseline characteristics with respect to age at the time of injury, last follow-up, injury side, sex, preexisting disease, ASA grade, AO classification, or Singh index in the HA group (45 patients [45.5%]) or the PFNA group (54 patients [54.5%]) before surgery.

**Table 1:** Baseline patient characteristics

Basic information		HA (***n*** = 45)	PFNA (***n*** = 54)	Statistical analyses	***P*** value
Gender	Male	15 (33.33%)	16 (29.63%)	χ^2^ = 0.157	0.69
	Female	30 (66.67%)	38 (70.37%)		
Age at the time of injury (years)		81.78 ± 8.18	80.52 ± 8.42	T = 0.7506	0.46
Age at last follow-up (years)		82.60 ± 8.21	82.00 ± 8.46	T = 0.3694	0.71
Side of fracture	Left	22 (48.89%)	28 (51.85%)	χ^2^ = 0.086	0.77
	Right	23 (51.11%)	26 (48.45%)		
AO classification	A1.1	11 (24.4%)	9 (16.67%)	χ^2^ = 7.458	0.28
	A1.2	16 (35.56%)	11 (20.37%)		
	A1.3	7 (15.56%)	13 (24.07%)		
	A2.1	7 (15.56%)	11 (20.37%)		
	A2.2	0 (0%)	4 (7.41%)		
	A2.3	0 (0%)	0 (0%)		
	A3.1	2 (4.44%)	3 (5.56%)		
	A3.2	2 (4.44%)	3 (5.56%)		
	A3.3	0 (0%)	0 (0%)		
ASA grade	I	6 (13.33%)	11 (20.37%)	χ^2^ = 1.313	0.73
	II	29 (64.44%)	33 (61.11%)		
	III	8 (17.78%)	9 (16.67%)		
	IV	2 (4.44%)	1 (1.85%)		
Singh index	I	1 (2.22%)	1 (1.85%)	χ^2^ = 5.221	0.27
	II	5 (11.11%)	16 (18.52%)		
	III	13 (28.89%)	12 (22.22%)		
	IV	14 (31.11%)	12 (22.22%)		
	V	12 (26.67%)	13 (24.07%)		
Comorbidities	Hypertension	18 (40.00%)	24 (44.44%)	χ^2^ = 1.198	0.66
	Cardiovascular and cerebrovascular disease	20 (44.44%)	17 (31.48%)	χ^2^ = 1.762	0.18
	Diabetes mellitus	12 (26.67%)	11 (20.37%)	χ^2^ = 0.546	0.46
	Chronic respiratory disease	12 (28.89%)	8 (14.81%)	χ^2^ = 2.909	0.09
	Neurological disease	9 (20.00%)	12 (22.22%)	χ^2^ = 0.073	0.79
	Thrombogenesis	6 (13.33%)	4 (7.41%)	χ^2^ = 0.949	0.33
	Urological disease	1 (2.22%)	4 (7.41%)	χ^2^ = 1.376	0.24

Table 1. The mean ± SD age at injury was 81.78 ± 8.18 years for the HA group and 80.52 ± 8.42 years for the PFNA group. The age at last follow-up was 82.60 ± 8.20 years in the HA group and 82.00 ± 8.46 years in the PFNA group. There were 50 left-sided and 49 right-sided fractures in total. The groups were similar in regard to sex (*P* = 0.69; χ^2^ = 0.157), AO classification (*P* = 0.28; χ^2^ = 7.458), ASA grade (*P* = 0.73; χ^2^ = 1.313), and Singh index (*P* = 0.27; χ^2^ = 5.221). The two groups had mostly similar rates of comorbidities including hypertension (*P* = 0.66; χ^2^ = 1.198), cardiovascular and cerebrovascular disease (*P* = 0.18; χ^2^ = 1.762), diabetes mellitus (*P* = 0.46; χ^2^ = 0.546), chronic respiratory disease (*P* = 0.09; χ^2^ = 2.909), neurologic disease (*P* = 0.79; χ^2^ = 0.073), thrombogenesis (*P* = 0.33; χ^2^ = 0.949), and urologic disease (*P* = 0.24; χ^2^ = 1.376). There was no difference in the basic underline diseases.

*Abbreviations*: *ASA* American Society of Anesthesiologists, *HA* Hemiarthroplasty, *PFNA* Proximal femoral nail antirotation

### Surgery data analysis

**Fig. 2:**
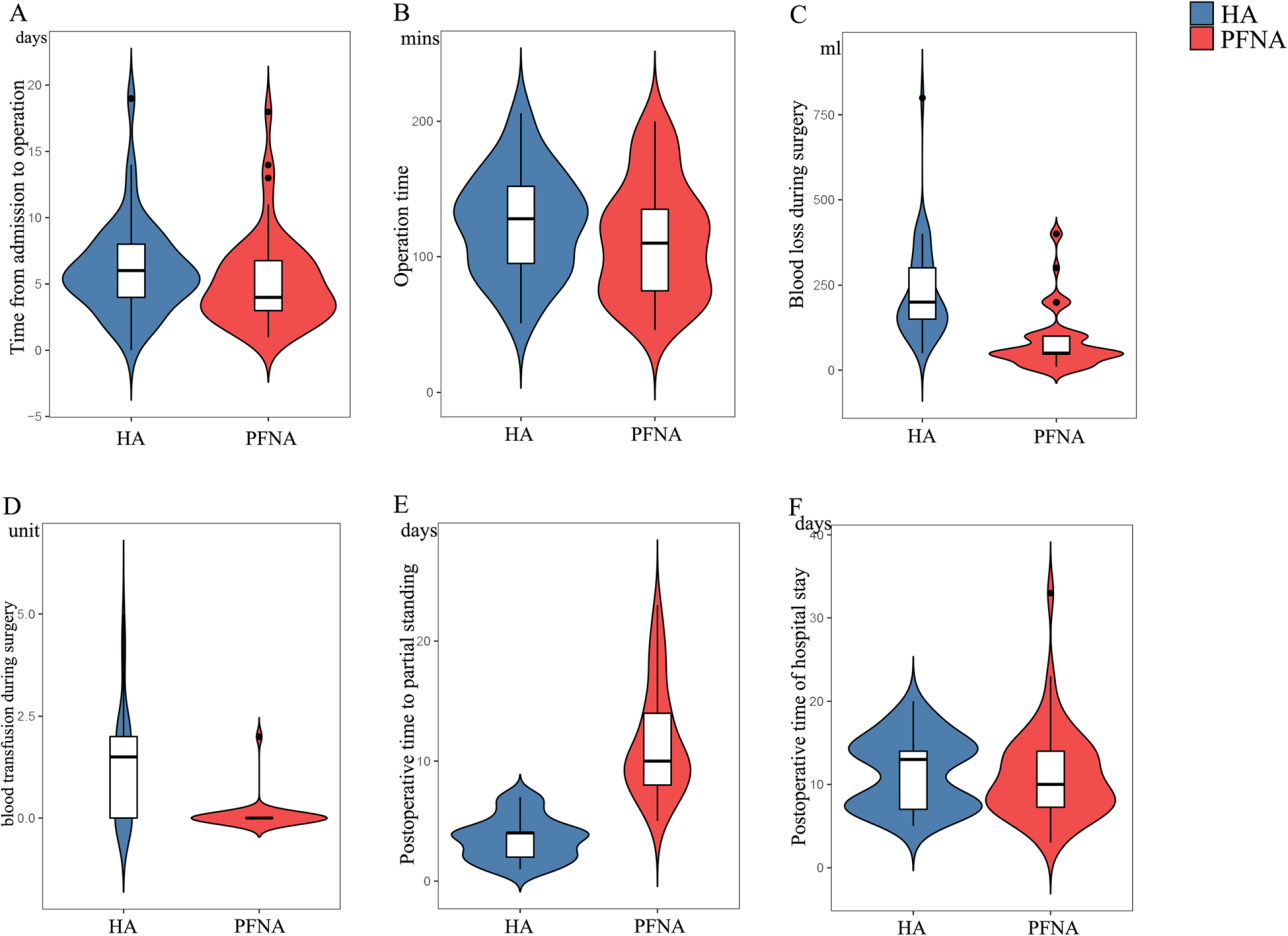
Comparisons of key patient data in Table 2. A Time from admission to surgery. B Surgical time. C Blood loss during surgery. D Blood transfusion during surgery. (E) Postoperative time to partial standing. F Postoperative duration of hospital stay. Abbreviations: HA, hemiarthroplasty; PFNA, proximal femoral nail antirotation

The results and analysis of the surgical data are presented in Table 2 and Fig. 2. Combined spinal and epidural anesthesia (CSEA) was the main anesthetic method used, and only one patient in each group was administered a local anesthetic. The surgical time did not differ between both groups. In addition, the median time from admission to surgery, postoperative length of stay, and total length of stay were all similar. Blood loss and subsequent transfusion during surgery were higher in the HA group than in the PFNA group; however, blood loss was < 500 mL. The HA group displayed early partial weight-bearing after surgery compared with the PFNA group. Majority of the patients in the HA group achieved partial weight-bearing within 10 days after surgery. More time was needed for patients who underwent PFNA to achieve partial weight-bearing. To further analyze the postoperative nutritional status and stress level, we performed routine blood tests at 1, 3, 5, and 7 days after surgery. Overall, postoperative hemoglobin and protein levels were similar for both groups, except the albumin level on postoperative day 5 (HA vs PFNA, 28.74 ± 4.15 g/L vs 30.31 ± 3.14 g/L) and white blood cell count on postoperative day 3 (HA vs PFNA, 9.47 ± 3.09 10^7^/L vs 8.03 ± 2.42 10^7^/L).

**Table 2:** Surgical data

Parameters		HA (***n*** = 45)	PFNA (***n*** = 54)	Statistical analyses	***P*** value
Median time from admission to surgery (days)		6 (4 ~ 8)	4 (3 ~ 7)	Wilcoxon W = 1464.5	0.08
Anesthesia method	General anesthesia	10	10	χ^2^ = 0.236	0.89
	CSEA	34	43		
	Local anesthesia	1	1		
Surgical time (min)		124.5 ± 37.92	112.5 ± 42.32	T = 1.466	0.15
Blood loss during surgery (ml)		200 (150 ~ 300)	50 (44.5 ~ 100)	Wilcoxon W = 2120.5	< 0.001
Blood transfusion during surgery (unit)		1.5 (0 ~ 2)	0 (0 ~ 0)	Wilcoxon W = 1821	< 0.001
Medium time to partial standing (days)		4 (2 ~ 4.5)	10 (8 ~ 14)	Wilcoxon W = 39	< 0.001
Postoperative mean hemoglobin level (g/L)	1 day	106.2 ± 20.24	99.13 ± 19.70	T = 1.778	0.08
	3 days	93.59 ± 21.71	90.69 ± 16.41	T = 0.760	0.46
	5 days	91.40 ± 14.78	92.87 ± 13.88	T = -0.510	0.61
	7 days	99.16 ± 15.52	95.41 ± 12.51	T = 1.331	0.19
Postoperative mean total protein level (g/L)	1 day	60.81 ± 8.18	58.413 ± 6.26	T = 1.651	0.10
	3 days	54.03 ± 6.49	53.57 ± 5.77	T = 0.377	0.71
	5 days	53.84 ± 6.75	54.88 ± 5.19	T = -0.863	0.40
	7 days	56.37 ± 6.04	57.08 ± 4.38	T = -0.677	0.51
Postoperative mean albumin level (g/L)	1 day	33.54 ± 5.39	33.56 ± 4.35	T = -0.016	0.99
	3 days	28.87 ± 3.98	29.66 ± 3.40	T = -1.083	0.28
	5 days	28.74 ± 4.15	30.31 ± 3.14	T = -2.147	0.04
	7 days	30.65 ± 3.56	31.89 ± 2.92	T = -1.913	0.06
Postoperative mean WBC (10^7^/L)	1 day	9.13 ± 3.25	8.70 ± 2.76	T = 0.708	0.48
	3 days	9.47 ± 3.09	8.03 ± 2.42	T = 2.596	0.01
	5 days	7.38 ± 3.09	7.91 ± 3.41	T = -0.805	0.42
	7 days	6.59 ± 2.24	6.67 ± 2.10	T = -0.195	0.85
Postoperative length of stay (days)		13 (7 ~ 14)	10 (7 ~ 14)	Wilcoxon W = 1322.5	0.45
Total length of stay (days)		18 (13 ~ 21.5)	15 (12 ~ 20)	Wilcoxon W = 1461.5	0.08

Table 2. No significant correlation was observed in the anesthesia method, operation time, median time from admission to operation, postoperative length of stay, and total length of stay between the HA and PFNA groups. The HA group showed more blood loss (*P* < 0.001; Wilcoxon W = 2120.5) and more blood transfusions (*P* < 0.001; Wilcoxon W = 1821) and required less time to achieve partial standing (*P* < 0.001; Wilcoxon W = 39) than the PFNA group. There was a difference in the mean albumin level on postoperative day 5 (*P* = 0.039; T = − 2.147) and the mean white blood cell count on postoperative day 3 (*P* = 0.013; T = 2.596). *Abbreviation*: *CSEA* Combined spinal and epidural anesthesia. *HA* Hemiarthroplasty, *PFNA* Proximal femoral nail antirotation

**Fig. 3:**
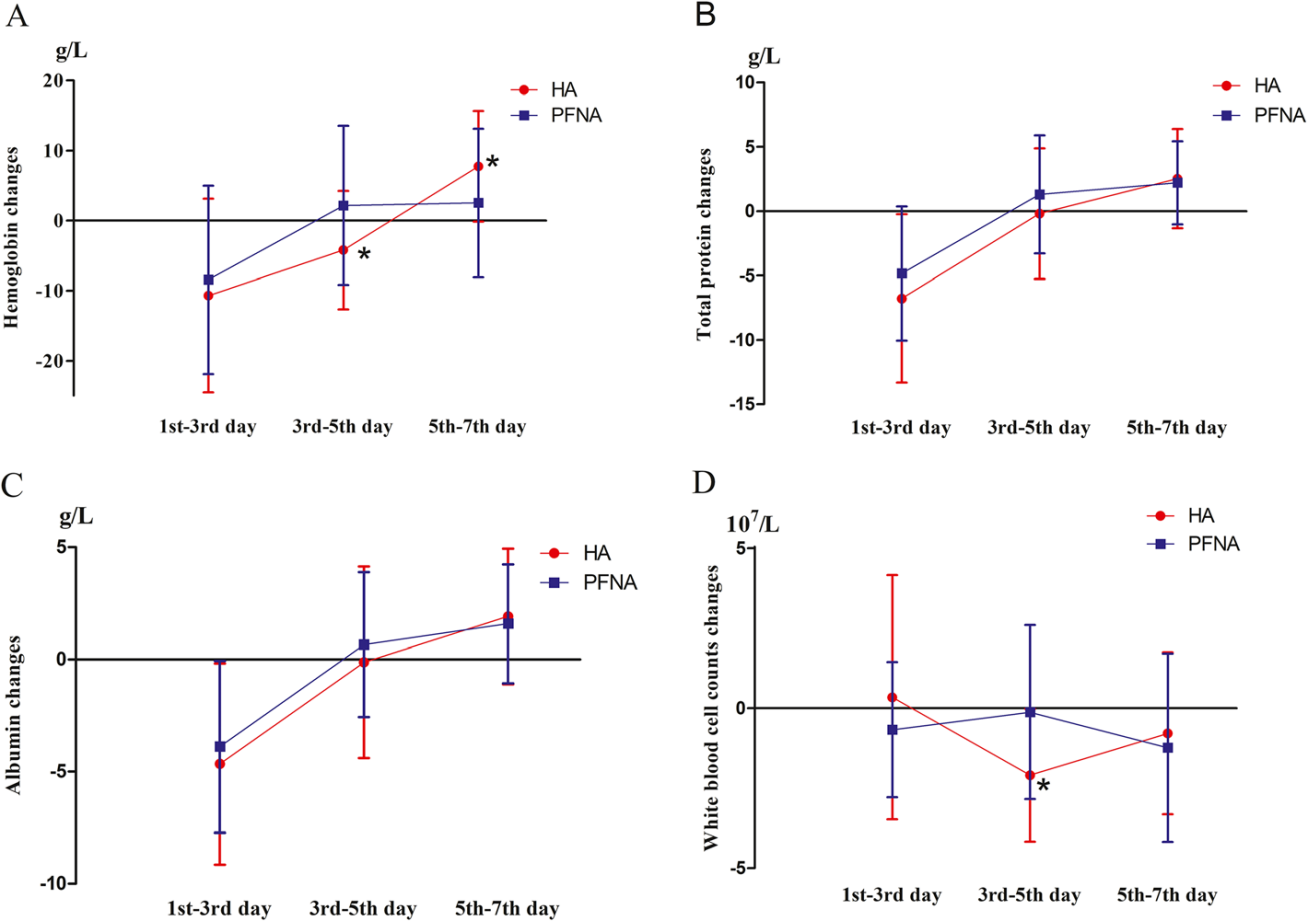
Postoperative change in the levels of hemoglobin, total protein, and albumin and white blood cell count. The levels of hemoglobin (Fig. 3**A**), total protein (Fig. 3**B**), and albumin (Fig. 3**C**) and white blood cell count (Fig. 3**D**) were measured on postoperative days 1, 3, 5, and 7. To follow the patient’s recovery over time, the changes from postoperative days 1 to 3, 3 to 5, and 5 to 7 were analyzed. The variation in the levels of hemoglobin, total protein, and albumin in the HA group on postoperative days 3 to 5 was negative; however, the results were positive in the PFNA group. During this period, the variation in hemoglobin level was − 4.20 ± 8.47 g/L for the HA group and 2.19 ± 11.36 g/L for the PFNA group (Fig. 3**A**, P^*^ < 0.05); the variation in the total protein level was − 0.19 ± 5.07 g/L for the HA group and 1.31 ± 4.59 g/L for the PFNA group; the variation in the albumin level was − 0.14 ± 4.27 g/L for the HA group and 0.65 ± 3.23 for the PFNA group; and the variation in the white blood cell count was − 2.10 ± 2.08 × 10^7^/L for the HA group and − 0.12 ± 2.72 × 10^7^/L for the PFNA group (Fig. 3**D**, P^*^ < 0.05). On postoperative days 5 to 7, the variations in the levels of hemoglobin, total protein, and albumin were positive. The variations in the hemoglobin level were 7.76 ± 7.90 g/L for the HA group and 2.54 ± 10.63 g/L for the PFNA group (Fig. 3**A**, P^*^ < 0.05); the variations in the total protein level were 2.53 ± 3.86 g/L for the HA group and 2.21 ± 3.23 g/L for the PFNA group; the variations in the albumin level were 1.91 ± 3.02 g/L for the HA group and 1.58 ± 2.66 g/L for the PFNA group; and the variations in the white blood cell count were − 0.78 ± 2.53 × 10^7^/L for the HA group and − 1.23 ± 2.94 × 10^7^/L for the PFNA group. The change in the levels of hemoglobin, total protein, and albumin and white blood cell count at other time periods showed no significance. Abbreviations: HA, hemiarthroplasty; PFNA, proximal femoral nail antirotation

As shown in Fig. 3, we analyzed the trend of postoperative blood test indicators. The variation in the levels of hemoglobin, total protein, and albumin in the HA group on postoperative days 3 to 5 was negative; however, the results were positive in the PFNA group. The variation in the levels of hemoglobin, total protein, and albumin in the HA group was positive on postoperative days 5 to 7. The marked changes in these indicators in the HA group were directly correlated to the early time of partial weight-bearing.

### HA group had higher Harris scores at 6-month follow-up

Recovery of hip function was evaluated using the Harris hip score questionnaire via telephone, WeChat, or outpatient visit at the postoperative 6-month follow-up visit, and the median scores for each item along with total scores are listed in Table 3. Harris hip scores at the 6-month follow-up were significantly higher in the HA group than in the PFNA group. The items with significantly better scores in the HA group included pain, walking support, walking distance, stairs, and hip joint mobility. Scores for other subitems, including limp, putting on shoes and socks, sitting time, entering public transportation, and deformity, were similarly distributed in both groups. No significant difference was evident in Harris grading. However, more patients in the HA group had scores in the good-to-excellent range than in the PFNA group. The preoperative, postoperative, and follow-up radiograph examinations and intraoperative findings are shown in Fig. 6.

**Table 3:** Harris hip scores at the postoperative 6-month follow-up

Characteristics	Subitem	HA (***n*** = 45)	PFNA (***n*** = 54)	Statistical analyses	***P*** value
Harris score	Total score	86.8 (81.9 ~ 90.23)	83.48 (75.13 ~ 88.23)	Wilcoxon W = 1536.5	0.02
	Pain	44 (40 ~ 44)	40 (40 ~ 41)	Wilcoxon W = 1468.5	0.04
	Limp	11 (9.5 ~ 11)	11 (10.25 ~ 11)	Wilcoxon W = 1266	0.92
	Walking support	7 (5 ~ 11)	7 (5 ~ 7)	Wilcoxon W = 1514	0.03
	Walking distance	8 (8 ~ 8)	6.5 (5 ~ 8)	Wilcoxon W = 1589.5	0.004
	Stairs	2 (2 ~ 3)	2 (1 ~ 2)	Wilcoxon W = 1496	0.03
	Wearing shoes and socks	2 (2 ~ 4)	2 (2 ~ 4)	Wilcoxon W = 1324.5	0.38
	Siting time	4 (4 ~ 5)	4 (3 ~ 5)	Wilcoxon W = 1332	0.38
	Public transportation	1 (1 ~ 1)	1 (1 ~ 1)	Wilcoxon W = 1282.5	0.44
	Deformity	4 (4 ~ 4)	4 (4 ~ 4)	Wilcoxon W = 1260	0.59
	Hip joint mobility	4.15 (3.85 ~ 4.35)	3.9 (3.64 ~ 4.15)	Wilcoxon W = 1612	0.005
Harris grade	Excellent (≥90 score)	13 (28.89%)	10 (18.52%)	χ^2^ = 5.155	0.16
	Good (80 ~ 89 score)	23 (51.11%)	22 (40.74%)		
	Fine (70 ~ 79 score)	5 (11.11%)	11 (20.37%)		
	Bad (< 70 score)	4 (8.89%)	11 (20.37%)		

Table 3. Harris hip scores at the 6-month follow-up were significantly higher in the HA group (HA group, 86.80 points; range, 81.9–90.23 points vs PFNA group, 83.48 points; range, 75.13–88.23; *P* = 0.024, Wilcoxon W = 1536.5). The scores for subitems, including pain (*P* = 0.045; Wilcoxon W = 1468.5), walking support (*P* = 0.026; Wilcoxon W = 1514.0), walking distance (*P* = 0.004; Wilcoxon W = 1589.5), stairs (*P* = 0.027; Wilcoxon W = 1496.0), and hip joint mobility (*P* = 0.005; Wilcoxon W = 1612.0), were significantly better in the HA group than in the PFNA group. Scores for other subitems, including limp, putting on shoes and socks, sitting time, entering public transportation, and deformity, were similar and similarly distributed in both groups. There was no significant difference in Harris grading. *Abbreviations*: *HA* Hemiarthroplasty, *PFNA* Proximal femoral nail antirotation

**Fig. 4:**
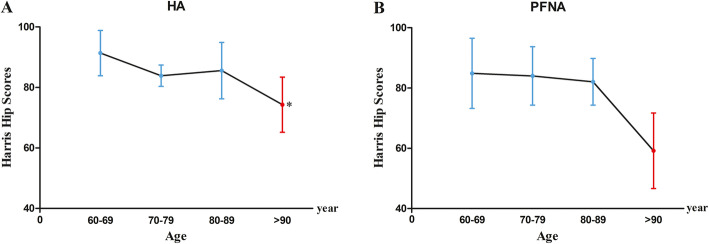
Effect of age on Harris hip scores 6 months after surgery. Patients were divided into subgroups according to age at surgery, and the resultant scores were as follows: there was a significant difference between the HA and PFNA groups in the subgroup of patients aged older than 90 years (Fig. 4A, P* < 0.05). Abbreviations: HA, hemiarthroplasty; PFNA, proximal femoral nail antirotation

In Fig. 4, we further analyzed Harris hip scores in each group by age. For the patients aged 60 to 69 years in the HA group, Harris scores were excellent in the HA group compared with those in the PFNA group. Harris hip scores decreased abruptly in patients aged older than 90 years, and this reduction was smaller in the HA group than in the PFNA group. The HA group had higher Harris scores than those patients older than 90 years in the PFNA group.

### Survival outcomes between two groups

**Fig. 5:**
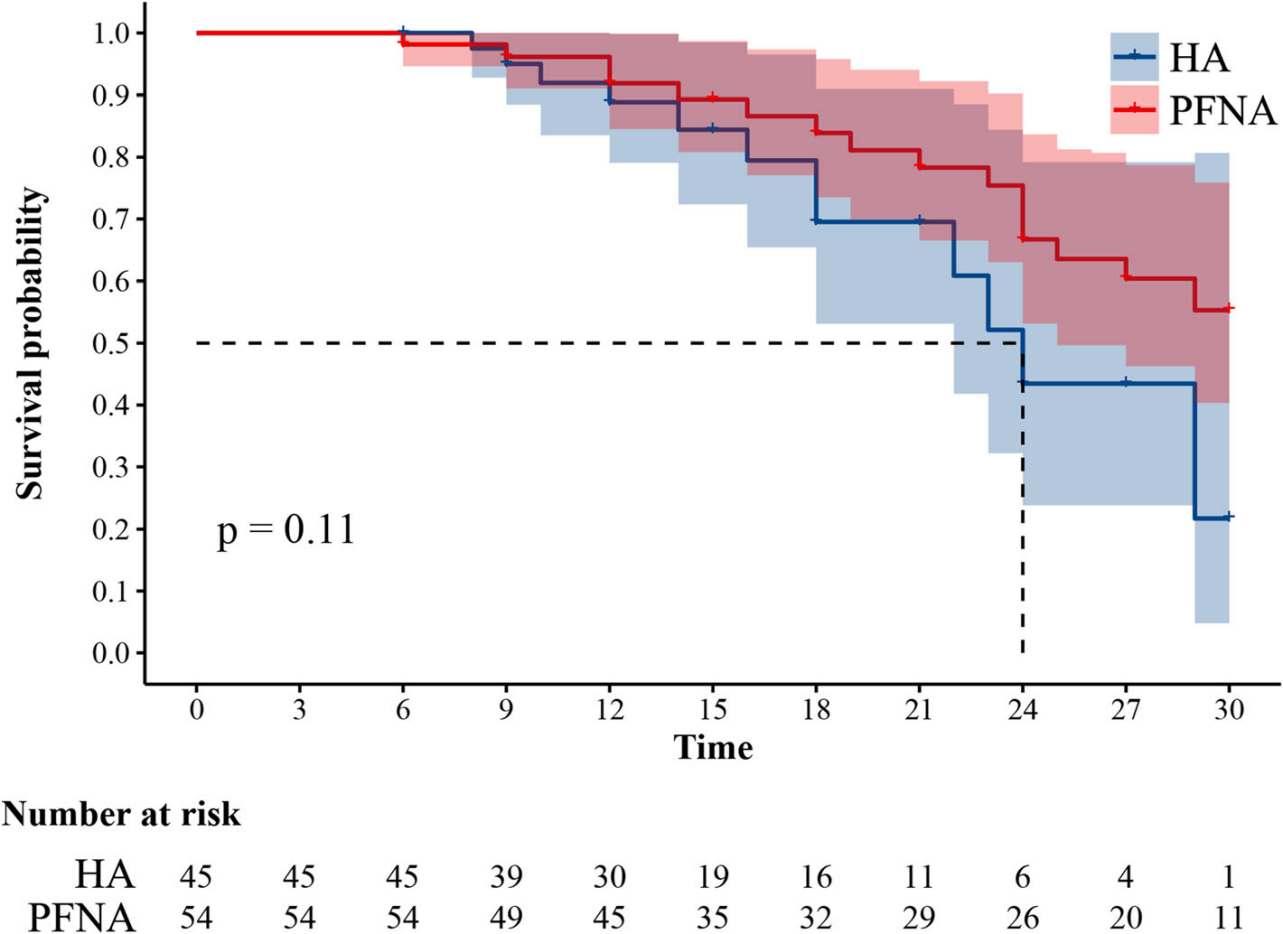
Kaplan-Meier survival plot. The blue line indicates the HA group; the red line indicates the PFNA group; *P* = 0.11. Abbreviations: HA, hemiarthroplasty; PFNA, proximal femoral nail antirotation

As of May 31, 2020, the total follow-up period for both groups was 30 months. Postoperative cumulative mortality data are shown in Table 4. At the time of the last follow-up, 12 patients (26.67%) in the HA group and 16 patients (29.63%) in the PFNA group had died. The mortality rate was 26.67 and 29.63% for the HA and PFNA groups, respectively. We did not observe a significant difference in the cumulative mortality rate of both groups at any follow-up time node. Kaplan-Meier survival curves are shown in Fig. 5; they confirmed that there were no differences in overall survival.

**Table 4:** Postoperative cumulative mortality in both groups

Mortality	HA (***N*** = 45)	PFNA (***N*** = 54)	Statistical analysis	***P*** value
9 Months	2 (4.44%)	2 (3.70%)	χ^2^ = 0.035	0.85
12 Months	4 (8.89%)	4 (7.41%)	χ^2^ = 0.073	0.79
15 Months	5 (11.11%)	5 (9.26%)	χ^2^ = 0.093	0.76
18 Months	8 (17.78%)	7 (12.96%)	χ^2^ = 0.443	0.51
21 Months	8 (17.78%)	9 (16.67%)	χ^2^ = 0.021	0.84
24 Months	11 (24.44%)	13 (24.07%)	χ^2^ = 0.002	0.97
27 Months	11 (24.44%)	15 (27.78%)	χ^2^ = 0.141	0.71
30 Months	12 (26.67%)	16 (29.63%)	χ^2^ = 0.106	0.74

Table 4. The postoperative cumulative mortality at each follow-up time point from 9 to 30 months after surgery. There were no differences between the two groups at follow-up. *Abbreviations*: *HA* Hemiarthroplasty, *PFNA* Proximal femoral nail antirotation

**Fig. 6:**
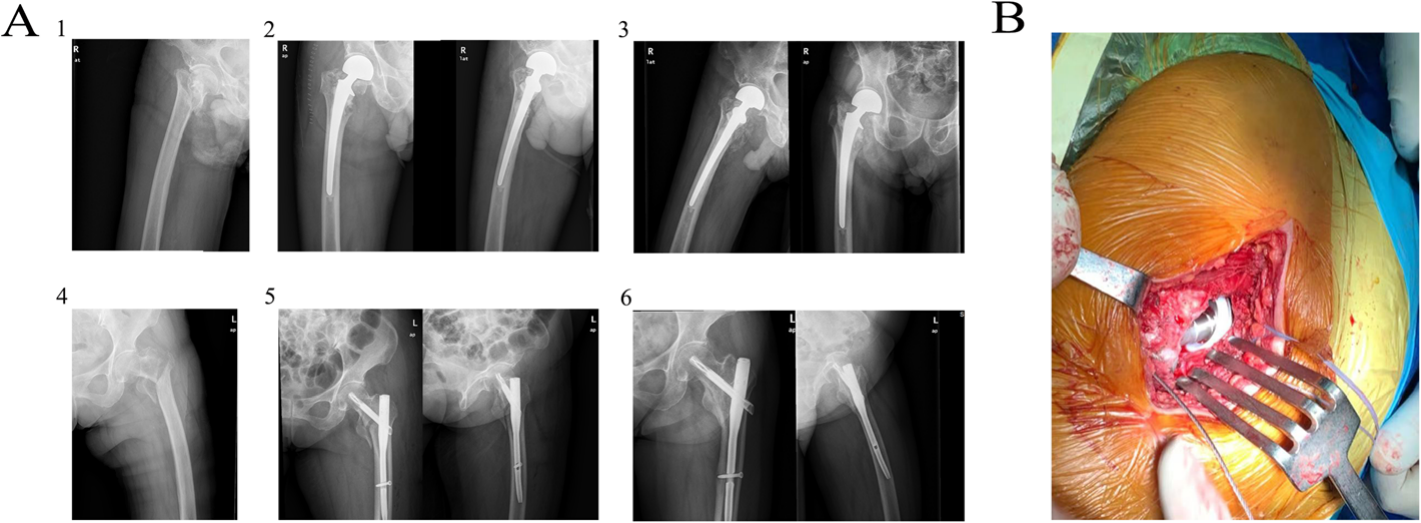
**A** (1) Preoperative images in patients receiving hemiarthroplasty (HA); (2) postoperative images of HA; (3) 6-month follow-up images of HA; (4) preoperative images in patients receiving proximal femoral nail antirotation (PFNA); (5) postoperative images of PFNA; (6) 6-month follow-up images of PFNA. **B** Intraoperative image of hip arthroplasty prosthesis

## Discussion

For elderly patients with IFF, the ultimate goal of treatment is to restore joint function as soon as possible and restore their mobility before trauma. Surgical treatment can restore the patient’s mobility as soon as possible, while non-surgical treatment makes the patient stay in bed for a long time, causing complications due to prolonged periods of bed rest. PFNA and HA, which are representatives of internal fixation and arthroplasty, respectively, have their own advantages in treatment characteristics. PFNA is more in line with the physiological and mechanical line of the femur and is more resistant to shear force and internal rotation and inversion deformity [15, 16]. Patients undergoing HA surgery can bear weight early after surgery, and HA is suitable for patients with osteoporosis or unstable IFF [17]. However, the current findings reflect a clinical dilemma. For PFNA, due to the inability of patients to bear body weight early in their recovery, a series of complications caused by prolonged bed rest may aggravate the patient’s underlying disease and affect postoperative recovery. This may be more severe than the trauma induced by surgery. For patients who underwent PFNA and have concurrent poor bone quality, bearing weight causes the spiral blade to cut the femoral head and neck [18]. HA allows patients to bear weight early in the recovery process and avoid bedridden syndrome. However, revision surgery for failed HA procedures is very difficult due to the trauma and financial burdens of a second surgery, and this is unbearable for many patients. In the present study, we provided a detailed explanation of the advantages and disadvantages of the two treatment options for patients and their families. The final treatment plan was determined by the patient or the authorized person.

The predominance of female patients in this study reflects the greater risk of trochanteric fractures in elderly women. This has been attributed to deficiency in endogenous estrogen and other aging-related changes in physiologic factors that increase the risk of osteoporosis and fractures in elderly women [19, 20]. In addition, in the case of severe osteoporosis, the spiral blade of PFNA may cause cutting injury of femoral head or femoral neck. To avoid such complications, the use of cement augmentation to reduce screw cortical cutting has been reported [21, 22]. Alternatively, arthroplasty can be used to avoid screw cutting.

For the A1.2 type of the IFF, the integrity of the greater trochanter is destroyed, the traction of the gluteus medius makes the anatomic reduction of the greater tuberosity very difficult, and there is greater risk of the intraoperative screws becoming loose [2]. There are also many difficulties during the HA procedure for repairing the A1.2 type of IFF. First, the greater trochanter is a surface marker for the posterolateral approach of hip arthroplasty. A dissociative greater trochanter may cause difficulties in identifying the surgical area. Second, conventional surgery for hip arthroplasty requires internal rotation and adduction of the lower limb to expose the hip joint capsule. However, if the greater trochanter is fractured, the internal rotation of the affected limb is restricted, resulting in poor exposure of the posterior lateral rotator muscles and joint capsule. We used the claw hook to adduct the greater trochanter to facilitate cutting off the lateral rotator muscles, thereby opening the joint capsule and subsequent osteotomy of the femoral neck. Third, as Gashi et al. reported in 2018, titanium cable or cementum can be used for greater trochanter fixation and intertrochanteric reinforcement [23].

The time between the admission and surgery is considerably long. During this time, we performed routine and preoperative examinations. We invited relevant departments for consultation to ensure the patient’s condition met the requirements of surgery and administration of anesthesia. There were some examinations that needed to be queued, such as 24-h dynamic electrocardiogram and electromyography of both lower limbs. There were still some preoperative interventions, such as regulating the blood sugar of diabetic patients and anti-infective intervention for patients with pneumonia or urinary tract infection. The above mentioned factors were the reason for the longer time between admission and surgery. However, after a series of preoperative examinations and interventions, the patients’ condition improved to a certain extent compared to when they were admitted to the hospital. Meanwhile, these efforts were for the smooth operation of the surgery and to avoid accidents while administering anesthesia. The mean surgical time for HA was longer than that for PFNA. This is consistent with results from both Zhou et al., who reported a longer surgical time in their arthroplasty group, and Özkayın et al., who reported that their internal fixation time was shorter [2, 24]. Surgical times may be affected by the complexity of arthroplasty to be performed and by the proficiency and cooperation of the surgical team. In addition, the time required to reduce the incidence of fracture is an important variable that cannot be standardized. The greater postoperative reduction in hemoglobin and albumin levels is also similar to the findings of a large multicenter study by Ekinci et al. that compared arthroplasty and internal fixation [23].

Most previous studies have not mentioned trends in postoperative hemoglobin and albumin recovery in HA vs internal fixation; therefore, they cannot effectively reflect the speed of stabilization after surgery. The rapid decrease in hemoglobin levels from postoperative days 1 to 3 in the HA group may be caused by blood loss during and after surgery. The same tendency can be seen in the total protein and albumin level. However, the white blood cell count increased from postoperative day 1 to 3 after surgery and the number was higher in the HA group than in the PFNA group. It is suggested that postoperative stress may have caused this. The downward trend observed in the levels of hemoglobin, total protein, and albumin in the HA group were suppressed from postoperative days 3 to 5, and they showed an upward trend from days 5 to 7 after surgery. The change during this period coincided with the time that the HA group started to bear partial weight after the surgery, which may have caused changes in the blood levels of these variables. These indexes returned to nearly preoperative values by postoperative day 7. It can be assumed that there was no significant difference in the nutritional status between the groups when the patients were discharged from the hospital. Early postoperative weight-bearing not only avoids bedridden complications but also promotes the improvement of the patient’s nutritional level and effectively combats postoperative stress. However, longer surgical times and higher blood loss during surgery are also traumatic to patients. In addition to blood transfusion, we used tranexamic acid to stop bleeding during the surgery. The use of tranexamic acid is of great significance to prevent excessive postoperative blood loss [25].

For the two groups of patients, we did not restrict the activities of the patients in the lying and standing positions. The data in Table 2 were recorded when the two groups of patients began to try partial weight-bearing. Although the patients in the PFNA group were not restricted in their activities after surgery, they started weight-bearing later than those in the HA group. Pain is a main problem that needs to be managed after surgery. More postoperative pain was found in patients in the PFNA group due to mismatch of the proximal end of the nail and the gluteus medius injuries at the point of nail injection [5, 26]. Even with effective analgesia, including oral or intravenous medications, some patients still could not tolerate postoperative incision pain. In this case, early weight-bearing may aggravate the pain experienced by the patients in the PFNA group, which results in the patients being unwilling to attempt early weight-bearing. Once the patient falls due to incision pain or other reasons while weight-bearing, the consequences become unbearable. Further, because of prolonged bed rest and surgical intervention, most elderly patients suffer from insufficient muscle strength within a short period of time after surgery. This situation can lead to instability of the affected hip joint, which makes the patient afraid to bear weight prematurely. Arthroplasty is recommended as a remedy for failure of internal fixation. Undergoing a second surgery in a short period of time is too burdensome for elderly patients [27]. Some patients will choose HA as a treatment plan to avoid the incision of nails during PFNA and restore joint function as soon as possible (with the purpose of achieving early weight-bearing).

The patients in the HA group had better Harris hip scores outcomes at the 6-month follow-up. They had better scores in walking support, walking distance, stairs, and hip joint mobility and better pain scores. The multicenter comparison by Ekinci et al. did not find significant differences in terms of the total Harris score after arthroplasty vs. fixation; however, certain sub-parameter scores were quite different, with patients reporting less pain and need for walking support and better walking distance and stair climbing after HA [23]. Our outcomes are contrary to those reported by Tang, who found that although the total Harris score was not different between the arthroplasty and PFNA groups, the fixation group had better scores in most of the sub-parameters, which suggests that PFNA ultimately allowed more social functionality [26]. PFNA surgery requires high preoperative traction and reduction, and the reduction effect directly influences postoperative recovery. HA requires high-quality prosthesis fixation, which is accomplished by division of the hip muscles, resulting in decreased strength of the lateral hip muscles and postoperative hip joint stability [28]. We found a marked decline in Harris scores after the age of 90 years in both groups (Fig. 4A, B). Ekinci et al. and Tang et al. have also reported declining Harris scores with age [23, 28]. Functional outcomes are influenced by many factors, including age, gender, underlying disease, social dependency, and postoperative complications [29].

However, no cases of postoperative infection were found in the two groups at the latest follow-up. The prevention of infection during HA is significant due to potentially catastrophic clinical outcomes. The use of image data alone may be not effective in diagnosing early infection. Falzarano et al [30] concluded that inflammation indicators (C-reactive protein, erythrocyte sedimentation rate, and procalcitonin) were good markers for arthroplasty infection screenings. If the infection can be detected within 4 weeks after the surgery, doctors can perform conservative plans before the microorganisms form biofilms. Rollo et al. also reported that using antibiotic-loaded spacer is a novel intra-articular anti-infection strategy [31].

Another important postoperative indicator to evaluate IFF repair among the elderly is the mortality rate. Luo et al. reported a 1-year follow-up mortality rate of 21.2% after bipolar HA compared to 11.3% after PFNA [29]. The mortality rate was 8.89% after HA and 7.41% after PFNA at the 1-year follow-up in our study. The most common reasons for death included underlying disease or trauma, such as heart failure, stroke, and traffic accident. At 6 months postoperatively, patient recovery of mobility can be ascertained via Harris scores, radiographic images, and outpatient evaluations. To date, we have not found a case of death directly caused by HA or PFNA among all the monitored patients.

The main screw used in PFNA lacks a trapezoidal cross section and caudal fork design. This prevents PFNA from transferring torque from the femoral head and neck to the cortical bone of the femoral shaft. This is an important reason why PFNA is not suitable for unstable IFF [18]. However, these advantages must be weighed against the risk of internal fixation-related complications including cut-out and collapse, which may be why some surgeons prefer HA in elderly patients. Opinions about HA for IFF have changed over time. A 2017 meta-analysis showed that arthroplasty can effectively reduce implant complications and probability of reoperation and suggested that arthroplasty was an attractive therapeutic option for IFF [32]. The intramedullary fixation system is prone to failure for unstable IFF or poor bone conditions; hence, HA should probably be the first choice of therapy in unstable and comminuted IFF in elderly patients [2, 33]. More importantly, once PFNA fails to manage unstable IFF, the trauma of a second surgery is unbearable for many patients.

There are several limitations to this study. It was a case-control study and had clear inherent limitations. We did not use randomization out of respect for the patient’s right of informed consent. We excluded patients with cognitive impairment, hemiplegia, and an inability to walk before their trauma, which may have caused a selection bias in the results. We initiated HA to treat IFF 3 years ago. Hence, the number of patients in the HA group who met the inclusion criteria may be small, and the follow-up period may be shorter. Large-scale randomized controlled trials or multicenter studies can be considered if necessary.

## Conclusion

In conclusion, the results of the study illustrated the advantage of HA for repairing IFF among the elderly. The advantages of HA included earlier weight-bearing, avoidance of complications such as long-term bed rest, and reduced risk of implant-related complications. The advantages of early weight-bearing in HA were significant in postoperative stress recovery. Patients who underwent HA also had better joint function at the 6-month follow-up, and there were no significant differences in interval mortality rates between the HA and PFNA groups during further follow-up. Therefore, we suggest that the benefit of HA can be considered in the clinical intervention of IFF among the elderly.

## Data Availability

The data in this study is not publicly available because it is derived from the patient electric records, surgical and nursing records. According to informed consent, we keep it confidential. If we obtain the approval from the institution, the data can be shared to researchers.

## Abbreviations

HA: Cemented hemiarthroplasty
PFNA: Proximal femoral nail antirotation
IFF: Intertrochanteric femoral fractures
AO: Arbeitsgemeinschaft für Osteosynthesefragen
ASA: American Society of Anesthesiologists score
HHS: Harris Hip Scores
